# Peptide Nucleic Acid Clamp‐Assisted Photothermal Multiplexed Digital PCR for Identifying SARS‐CoV‐2 Variants of Concern

**DOI:** 10.1002/advs.202306088

**Published:** 2024-01-19

**Authors:** Lexiang Zhang, Rokshana Parvin, Siyue Lin, Mingshuo Chen, Ruixuan Zheng, Qihui Fan, Fangfu Ye

**Affiliations:** ^1^ Joint Centre of Translational Medicine the First Affiliated Hospital of Wenzhou Medical University Wenzhou Zhejiang 325035 China; ^2^ Oujiang Laboratory (Zhejiang Lab for Regenerative Medicine, Vision and Brain Health); Wenzhou Institute University of Chinese Academy of Sciences Wenzhou 325000 China; ^3^ Key Laboratory of Structural Malformations in Children of Zhejiang Province the Second Affiliated Hospital and Yuying Children's Hospital of Wenzhou Medical University Wenzhou Zhejiang 325027 China; ^4^ Department of Biomedical Engineering Columbia University New York NY 10027 USA; ^5^ Beijing National Laboratory for Condensed Matter Physics Institute of Physics Chinese Academy of Sciences Beijing China 100190

**Keywords:** digital PCR, droplet microfluidics, single nucleotide polymorphisms, peptide nucleic acid, SARS‐CoV‐2 variant

## Abstract

The unprecedented demand for variants diagnosis in response to the COVID‐19 epidemic has brought the spotlight onto rapid and accurate detection assays for single nucleotide polymorphisms (SNPs) at multiple locations. However, it is still challenging to ensure simplicity, affordability, and compatibility with multiplexing. Here, a novel technique is presented that combines peptide nucleic acid (PNA) clamps and near‐infrared (NIR)‐driven digital polymerase chain reaction (dPCR) to identify the Omicron and Delta variants. This is achieved by simultaneously identifying highly conserved mutated signatures at codons 19, 614, and 655 of the spike protein gene. By microfluidically introducing graphene‐oxide‐nanocomposite into the assembled gelatin microcarriers, they achieved a rapid temperature ramping‐up rate and switchable gel‐to‐sol phase transformation synchronized with PCR activation under NIR irradiation. Two sets of duplex PCR reactions, each classifying respective PNA probes, are emulsified in parallel and illuminated together using a homemade vacuum‐based droplet generation device and a programmable NIR control module. This allowed for selective amplification of mutant sequences due to single‐base‐pair mismatch with PNA blockers. Sequence‐recognized bioreactions and fluorescent‐color scoring enabled quick identification of variants. This technique achieved a detection limit of 5,100 copies and a 5‐fold quantitative resolution, which is promising to unfold minor differences and dynamic changes.

## Graphical abstract

PNA‐assisted NIR photothermal multiplexed dPCR technique recognizes SNPs at codons T19R, O614G, and H655Y of the spike protein gene, thereby discriminating Omicron and Delta variants from the wild‐type of SARS‐CoV‐2. Gelatin microcarriers loaded with GO nanocomposite assist PCR thermocycling under NIR irradiation, where three PNA probes target against SNPs to selectively amplify mutated templates via replication with multi‐color labeling.

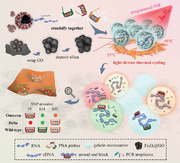

## 1 Introduction

The rapid accumulation of mutations in the genome of the severe acute respiratory syndrome coronavirus type 2 (SARS‐CoV‐2) has led to numerous variants with distinct phenotypes, posing significant challenges to public health systems.^[^ ^1^, ^2^ ^]^ More specifically, the original SARS‐CoV‐2 vaccine has been less effective against Delta variants, and Omicron variants have shown even increased infection rates.^[^ ^3^, ^4^ ^]^ For better disease control, a wide range of biotechnological approaches have been explored to distinguish these variants by uncovering the underlying nucleotide polymorphisms and diversity.^[^ ^5^, ^6^ ^]^

Currently, the most widely accepted method of next‐generation sequencing still faces challenges in detecting variants at the population level over the long term. Various types of clustered regularly interspaced short palindromic repeats/associated Cas protein (CRISPR/Cas) ‐mediated assays that can adapt cleavage preferences of Cas enzymes have been used to distinguish highly prevalent mutation of L5F, D80A, D215G, R246I, K417N, L452R, Y453F, etc.^[^ ^7^ ^]^ Despite their fast turnaround time, these assays are usually hampered by a necessary but separate preamplification step. The cost and practicability may limit the accessibility of these existing assays.^[^ ^8^ ^]^ Polymerase chain reaction (PCR) has been reported for genotyping variants by comparing the melting temperature shift with standard samples, which was caused by alternation in purine and pyrimidine bases. However, the detection could be challenging at times due to low single‐to‐noise ratios.^[^ ^9^, ^10^ ^]^ Isothermal amplification assays, such as recombinase polymerase amplification and loop‐mediated isothermal amplification, provided rapid and low‐cost alternatives, while they were often subject to nonspecific amplification, resulting in false‐positive results.^[^ ^11^, ^12^ ^]^

Introducing peptide nucleic acids (PNAs) into PCR can be a promising alternative method. PNAs are DNA analogues that feature a neutral N‐(2‐aminoethyl) glycine linkage in place of negatively phosphate deoxyribose backbone.^[^ ^13^ ^]^ The thermal stability of the PNA/DNA duplexes is stronger than that of double‐stranded DNA and DNA/RNA hybrids due to the absence of charged phosphate groups and resulting electrostatic repulsion in PNA's structure. More importantly, the formation of PNA/DNA duplexes is strongly affected by mismatches of even one single base pair.^[^ ^14^ ^]^ This unique property is responsible for its remarkable capacity of detecting gene expressions and low‐prevalent mutant alleles with high sensitivity and simplicity, particularly in microarrays and biosensors.^[^ ^15^ ^]^ Thus, the hybridization of PNA to target DNA holds great promise for discriminating single‐nucleotide polymorphisms (SNPs). Furthermore, different variants are often characterized by crucial SNPs at multiple loci. Pooling SNPs together in a bulk real‐time PCR solution increases the interference among different template during amplifications, while using multiple testing tubes and facilities is less preferable if considering applicability. Therefore, applying the PNA clamping technique to resolve SNPs in a multiplex‐friendly and high‐throughput mode becomes highly desirable to facilitate the rapid recognition of virus variants.

Meanwhile, droplet‐based digital PCR (ddPCR) has been built on separating PCR reaction solution into numerous droplet compartments, so that single molecules can be amplified and visualized. This approach offers several advantages, such as high sensitivity due to increased local template concentrations, and absolute quantification through its “digital” binary readout.^[^ ^16^ ^]^ Therefore, ddPCR has rapidly gained widespread adoption for detecting subtle differences and dynamic changes of nucleic acid sequences, as well as rare events that may be obscured by a high abundance of the dominant type.^[^ ^17^, ^18^ ^]^ Typically, ddPCR could handle dozens of subtypes of classification only via 4‐color‐fluorophore labeling, ultimlately achieving attomolar sensitivity.^[^ ^19^, ^20^ ^]^ Therefore, microfluidic droplets appear to be an ideal modality for simultaneous labeling of the multiple targets.^[^ ^21^, ^22^, ^23^ ^]^ Nevertheless, the high costs and complex facilities restricted the widespread deployment of ddPCR for large‐scale applications. Generally, to achieve the multistage temperature cycling required for PCR, various thermal heating approaches have been employed, including Peltier, oil baths and microwave, while they demanded a large thermal capacitance for the entire system, encompassing reaction volumes, well plates and metal heating blocks. Recently, both our group and others have demonstrated that the inclusion of 2D nanomaterials, e.g. graphene oxide (GO), in microcarriers,^[^ ^24^, ^25^, ^26^ ^]^ enabled precise temperature control of PCR microcarriers or substrate chips via NIR‐controlling module,^[^ ^27^, ^28^, ^29^ ^]^ overcoming the dependance on expensive and bulky PCR infrastructure. They offer excellent thermal conductivity and great potential to reduce the heat capacity and energy consumption by confining these effects solely to the reaction volumes.

**Figure 1:**
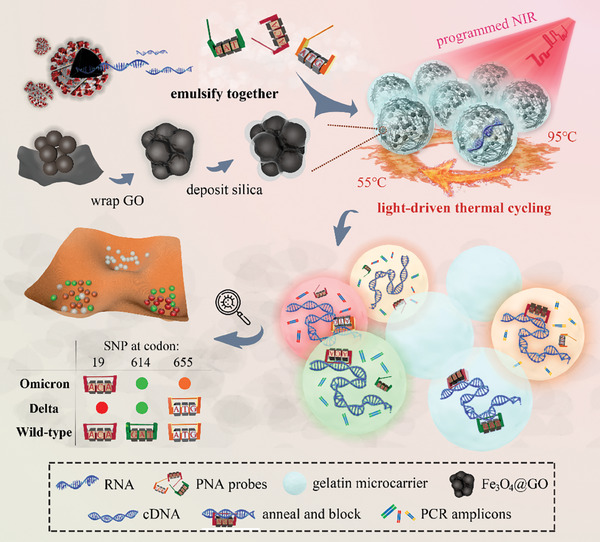
Schematic of the PNA‐assisted NIR photothermal multiplexed dPCR. The system can recognize SNPs at codons T19R, O614G, and H655Y of the spike protein gene, thereby discriminating Omicron and Delta variants from the wild‐type of SARS‐CoV‐2. Gelatin microcarriers loaded with GO nanocomposite assist PCR thermocycling under NIR irradiation, where three PNA probes target against SNPs to selectively amplify mutated templates via replication with multi‐color labeling.

In this paper, we present a novel NIR‐driven dPCR microcarriers that incorporated triple PNA‐clamps and fluorescing color codes for Omicron and Delta variants discriminations (**Figure** **1**). We expect that the microcarriers integrated with PNA and GO could be endowed with point mutation discrimination features, and the NIR controllable heating capability could greatly facilitate the thermocycling and its accessibility. We adhered the GO nanosheet to the surface of nanoparticle clusters, followed by the deposition of a silica layer to confer uniform dispersion and good biocompatibility to the resulting nanocomposite. Microcarriers containing reagents and nanocomposite were microfluidically‐fabricated with temperature‐sensitive gelatin substrate, which underwent sol‐to‐gel phase transition synchronously with PCR process. In this case, reactions of harbored nucleic acid molecules were promoted in the liquid‐state microcarriers, and the post‐PCR microcarriers were solidified into microgels to benefit the subsequent rinsing process and long‐term storage. Owing to the NIR heating capacity of GO, the obtained microcarriers could effectively facilitate the controlled dPCR process by NIR irradiation. Moreover, three different PNA molecules, each with its corresponding fluorescent‐color probes, were encapsulated with viral DNA to screen for SNPs at codons T19R, O614G, and H655Y. The PNAs paired with targeted wild‐type sequences and blocked their extension, functioning as discriminators to selectively amplify any mutation present in the inspected regions. These results suggest that the PNA‐assisted photonic ddPCR is a promising technique for detection of SARS‐CoV‐2 variants, as well as subtypes of bacteria and tumors, providing a portable, sensitive, and cost‐effective platform.

## 2 Results and Discussion

### 2.1 Assembly and Characterization of Photothermal Responsive Microcarriers

**Figure 2:**
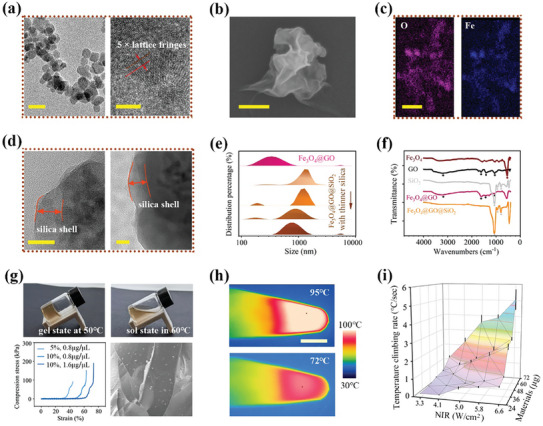
Assembling and physicochemical characterization of the Fe_3_O_4_@GO@SiO_2_ nanocomposite‐doped hydrogel. a) High‐resolution TEM images of Fe_3_O_4_ nanoparticles indicating the single‐crystalline state. b) SEM image of Fe_3_O_4_@GO composite. c) Fe and O elemental mapping analysis corresponding to the morphology. d) TEM images of silica deposit as the outside shell of Fe_3_O_4_@GO@SiO_2_. e) Changes in the size distribution of nanocomposites along the assembly steps, particularly influenced by the duration of silica deposit. f) FTIR spectra of each composition and their assembly system. g) Thermal‐reversible gel‐sol transition of the sample. Mechanical behavior of compression stress‐strain curves of gelatin with different concentrations. The legend values indicate concentrations of gelatin and composite, respectively. SEM images show the nanocomposite scattering in the gels. h) Thermal images of NIR‐heating sample at different PCR temperature stages. i) Temperature ramping up rate under various NIR irradiation intensities and nanomaterial loading amounts. Error bars, mean ± s.e.m. (*n* = 3). Scale bar, 25 nm in (a left, d), 5 nm in (a right), 150 nm in (b), 1 µm in (c), 3 mm in (h), and 50 µm in (g).

Generally, GO‐loaded nanocomposite was constructed by wrapping Fe_3_O_4_ NPs with GO and the following outside silica deposition as previously reported.^[^ ^27^ ^]^ Fe_3_O_4_ NPs have been successfully synthesized via a hydrothermal method. High‐resolution TEM analysis showed highly symmetrical lattice fringes of ≈0.296 nm, affirming the single crystalline nature of the obtained NPs (**Figure** **2**a). The GO nanosheets were fabricated by sonication exfoliation from the bulk GO crystals. XRD and Raman characterizations of GO nanosheets confirmed the successful construction (Figure S1a–c, Supporting Information). Electron images (Figure 2b) show that the Fe_3_O_4_@GO nanomaterials had a morphology with thin lamellar covering onto dozens of nanospheres, with a typical diameter of ≈400 nm. The size of the nanocomposite was reduced by half compared to previous work, aiming to increase the specific surface area and enhance the photothermal efficiency consequently. Since they adhered onto NPs through dopamine, the flexible GO nanosheets could prevent agglomeration caused by van der Waals force, which is beneficial for achieving uniform heating of the microcarriers (Figure S1d, Supporting Information). Furthermore, the profiles of Ferrum (Fe) and Oxygen (O) elements matched well with the morphology of the nanomaterials observed in the SEM (Figure 2c), indicating that the expected hierarchical structure has been obtained.

To ensure the feasibility of NIR‐mediated PCR, it is crucial to completely coat GO with biocompatible materials to prevent its adsorption with nucleic acid molecules. This was assessed by evaluating its impact on PCR product bands through gel electrophoresis (Figure S2a, Supporting Information). Considering that excessive silica deposition hindered photothermal responsiveness and led to the formation of inhomogeneous clods, we optimized the parameters of Fe_3_O_4_ size, the input of silica source, the stirring duration, and added continuous ultrasonic agitation. Through these optimizations, we successfully constructed a very thin and complete layer, ensuring improved homogeneity in heating and imaging (Figure 2d). In details, the thickness of silica could be modulated by controlling the tetraethoxysilane input and stirring time while all other conditions remained constant in the Stöber synthetic route. In this case, the obtained nanocomposite size could be tuned from 1313 to 742 nm (Figure 2e). Polydispersity indexes and standard deviations of generated composites’ size are provided in Table S1 (Supporting Information). A thin layer of 70 nm was preferred to maximize the efficiency of internal GO to absorb NIR. Meanwhile, by comparing the Fourier transform infrared (FTIR) spectra of the hierarchical nanocomposites with that of the elemental compositions (Figure 2f), the disappeared absorption peaks 1060 cm^−1^ of GO and 546 cm^−1^ of Fe‐O in Fe_3_O_4_ confirmed the entire coverage of the SiO_2_ shells.

Subsequently, we employed low‐gelling point (≈55 °C) gelatin as the substrate base to improve mechanical property compared to aqueous microdroplet in solution state. Additionally, we doped the Fe_3_O_4_@GO@SiO_2_ nanocomposite to impart intelligent responsiveness. As temperature increased, the storage moduli G′ of obtained gel dropped greatly until intersected with the loss modulus curve at ≈55 °C, implying the occurrence of the phase transition (Figure 2g). Notably, this thermosensitive phase transition could be reversible. The hydrogel polymers melted and broke down into short chains within the PCR temperature range. This process not only ensures adequate molecular diffusion, but also provides sufficient support for dispersing the nanocomposite effectively. To gain these benefits, 5–10 wt/v % gelatin concentrations were chosen from a range of 1–35% (Figure S2b, Supporting Information). If the concentration exceeds the optimized level, the increased viscosity significantly hinders amplification efficiency, as evidenced by the weakened product band. Another benefit of using gel substrates was that these gelatin droplets can solidify into microbeads after PCR, effectively preserving the monoclonal information without concerns of droplet merging. Moreover, composite gels with gelatin concentrations between 5–10% exhibited compression strength of 60–80 kPa at a breaking compressive strain of 40–70%, rendering them flexible enough to endure subsequent rinsing processes.

The high NIR‐to‐heat conversion ratio of GO nanosheets makes the composite gels highly responsive to NIR irradiation. We investigated the phase changes in response to real‐time temperature change, considering the variations of nanomaterials load and NIR laser (808 nm) intensities. The results depicted in Figure 2h,i showed a rapid heating rate of over 4 °C per second. The temperature reached 95 °C within 40 s of NIR exposure, and it took only 8 s to rise from 68 to 95 °C. In contrast, the photothermal effect of pure gelatin and Fe_3_O_4_@SiO_2_‐doped gelatin under the same irradiation condition was negligible (Figure S3, Supporting Information). More importantly, this effective photothermal responsiveness exhibited excellent properties for maintaining a constant temperature at specific PCR stages by controlling NIR intensity. Compared to previous work,^[^ ^30^ ^]^ the optimized system, featuring with smaller nanomaterial size and thinner shell, demonstrated more efficient and controllable responsiveness, while also preventing material sedimentation within the microcarriers.

**Figure 3:**
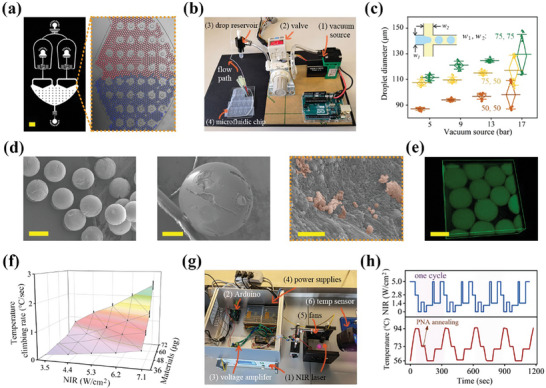
Fabrication of microcarriers with different responsiveness for dPCR thermocycling. a) Dual flow‐focusing configurations emulsified two reactions separately and collected together for analysis. Red and blue inks were added to measure the size uniformity. b) Device integrated with vacuum pressure suction module to fabricate droplets. c) Microcarrier diameter and uniformity as functions of vacuum pressure and junction dimension. d) SEM images showing morphology of microcarriers after supercritical drying, where the nanocomposite can be seen dispersing in the gel. e) Fluorescence confocal image showing FITC‐labeled nanocomposite outlining the shape of microcarriers. f) The heating rate of the microcarriers varied smoothly with the NIR intensity and nanomaterials loading amount. Error bars, mean ± s.e.m. (*n* = 3). g) Experimental flowchart of homemade automated NIR radiation module that mediates thermal cycling of photothermal PCR. h) 4 repetitive PNA‐assisted PCR temperature cycles achieved by calibrated NIR output programs. Scale bar, 500 µm in (a), 200 µm in (d left), 50 µm in (d middle), 1 µm in (d right), and 100 µm in (e).

To examine SNPs at the single‐molecule level, responsive microcarriers for dPCR were fabricated by introducing the previously mentioned reaction premix and oil phase into a co‐flow structured microfluidic device (**Figure** **3**a). The two oil streams vertically partitioned the composite solution stream into water‐in‐oil emulsions. To integrate microfluidic operations and manage the small liquid volume effectively, a customized vacuum pressure regulation module was connected to the channel outlet. This module draws the fluids in and forms droplets at a production frequency of ≈10^3^ Hz under ‐17 kPa vacuum source (Figure 3b). We found that the diameter of microcarriers was primarily determined by dimensions of the microchannels, such as the width and cross section depth, and was minimally influenced by biphasic flow rates (Figure 3c; Figure S4, Supporting Information). A typical droplet array with a diameter of 116 µm can ensure sufficient detection sensitivity. This size was utilized in this study, corresponding to ≈61200 droplets generated from a 50 µL solution.

Monodispersity of the microcarriers is crucial for accurate and absolute quantification in dPCR. To characterize this parameter, gel microbeads were dried with supercritical ethanol condition to prevent shape collapse before electron microscope observation. Typically, gel microbeads underwent a gradual soaking process, with each step lasting half an hour in ethanol solutions of increasing concentrations: 50%, 60%, 70%, 80%, 90%, and finally 100%. Subsequently, the samples were subjected to supercritical CO_2_ drying at 10 °C for 3 h. The electron images (Figure 3d) validated the well‐performed uniformity, sphericity, and efficient nanomaterial loadings in the representative morphology. The microbead retained its uniform shape and single‐molecule information in water environment and was found to swell to twice its original size after 3 h (Figure S5, Supporting Information). Subsequently, the silica shell of nanocomposite was labelled with fluorescein isothiocyanate (FITC) and examined using a confocal microscopy, revealing their stable and uniform distribution in the gelatin microbeads (Figure 3e; Figure S6, Supporting Information).

The NIR‐driven heating and temperature control of the microcarriers was examined by tuning key parameters, such as NIR intensity and nanocomposite concentration, from 3.5 to 7.1 W cm^−2^ and 36 to 72 µg, respectively. The results showed that all investigated microcarriers were able to reach 95 °C quickly and yielded a relatively uniform temperature field, as indicated by in situ infrared thermal imaging (Figure 3f). To simply and accurately repeat the irradiations, a custom NIR remote control source was employed to automatically manage the PCR thermal cycles (Figure 3g). The essential concept is to linearly map the range of NIR power to the range of the input virtual signal at the I/O interface, and then orchestrate the thermal cycling by assigning functions of NIR intensity and duration. In our repeated PCR cycles, an additional 75 °C stage for PNA annealing were added prior to primer annealing. Notably, microcarriers exhibited high sensitivity to NIR on/off and increasing/reducing conditions, maintaining an essentially constant responsiveness over four cycles of light illumination (Figure 3h). The Arduino circuit chip and embedded program activated three pairs of fans when the NIR was turned off, increasing cooling speed from 0.5 °C s^−1^ under natural cooling conditions to 1 °C s^−1^. Taking together, these results presented the repeatable and controllable NIR‐responsive performance of dPCR microcarriers. Compared to the conventional methods employing external heaters, this NIR approach features heating accomplished by heat sources inside the solution, which offers advantages such as low energy consumption, non‐contact heating, and ease of miniaturization and integration.

### 2.2 PNA Clamp‐Assisted PCR Strategy for Profiling SNP Signatures

**Figure 4:**
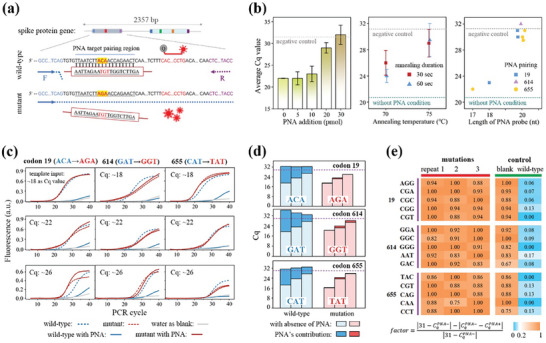
Principle and specificity of PNA design to distinguish SNPs at codon 19, 614, and 655 of the spike protein gene. a) Design of the PNA‐assisted assay for detection of point mutation at codon 19. A PNA sequence was introduced to block the amplification of wild‐type allele, and a TaqMan assay was included as amplification reporter. b) PNA clamping effect on wild‐type DNA as functions of loading amount, annealing temperature and duration, and sequence length. mean ± s.e.m. (*n* = 3). c) qPCR fluorescence was measured at three codons with different DNA inputs indicating that PNA clamps blocked wild‐type templates amplification while had negligible impact on mutant templates. d) Apparently different contributions of PNA on wild‐type and mutant templates amplification across those samples. The blank level indicated by the dotted lines represented the mean + twofold of the s.e.m. from three negative control tests without DNA input. e) Heatmap showing PNA‐assisted qPCR enabled the identification of 15 other possible mutations corresponding to the T19R, O614G, and H655Y codon changes. A factor is defined to describe the relative effect of PNA on the testing samples, where the value of 31 in the equation stands for the Cq of negative control.

Based on the SARS‐CoV‐2 genetic lineages tracking systems funded by NIH and its collaborators, typical hot spot mutation sites have been utilized to identify specific viral variants through various biotechnologies.^[^ ^31^, ^32^ ^]^ In this study, we applied the proposed strategy to identify the signatures T19R, D614G, and H655Y of spike protein to distinguish Delta and Omicron variants from wild‐type. These SNPs were highly conserved in >98.6%, >99.8%, >99.7% of variant‐specific sequences, respectively (**Figure** **4**a; Figure S7, Supporting Information). Theoretically, the results from either of the two combinations can achieve lineage differentiation with an accuracy exceeding 98.3%. Of note, considering PNA covers a dozen of nucleotides (nt), targeted regions have been carefully chosen to exclude any other common mutations.

The change of amino acid T/R, O/G and H/Y mainly corresponded to ACA to AGA substitution at codon 19, GAT to GGT substitution at codon 614, and CAT to TAT substitution at 655, respectively. Accordingly, the PNA clamps were designed to be reversely complementary to the wild‐type sequences. PNA clamping efficiency and selectivity were subsequently tested using wild‐type and mutant templates in qPCR with an extra binding step to facilitate PNA pairing. If paired, PNA would tightly clamp onto the DNA whenever it desaturates to single‐stranded molecules at 95 °C, blocks PCR primers extension and pauses the duplications. To this end, various key factors, including concentration, annealing temperature and duration, and nucleotide length, of the PNA clamps were explored to enhance the synergies (Figure 4b). Specifically, the samples added more than 30 pmol of PNA resulted in a significantly increased Cq value compared to the level of the negative control, whereas 5–30 pmol addition brought a trend of increasing Cq with less pronounced growth. This was attributed to insufficient amount of PNA to clamp onto all DNA templates. The amplification was dramatically suppressed, and the outcome reached a plateau at blank sample levels when the PNA annealing temperature was set to 5 °C above its own Tm and incubation exceeded 10 s during thermocycling. Additionally, it was revealed that PNAs shorter than 20 nt in length could barely inhibit DNA amplification of wild‐type, while its blocking efficiency was optimized once the length reaches 20 nt. Based on these results, several applicable designs that place the mutation site in the middle region have been demonstrated in Table S2 (Supporting Information).

Then, the experiments with samples of wide concentration gradients (Cq values ranged ≈18, 22, and 26) were conducted under the optimized conditions (Figure 4c,d). As expected, amplifications of all wild‐type samples spiked with PNA were inhibited, resulting in weak fluorescent patterns, and their Cq values were similar to those of the blank samples. In comparison, mutant DNA displayed normal amplification curves after adding PNA, indicating that designed PNA selectively blocked DNA amplification with wild‐type sequences. In addition to mutations with the most reported sequences tested earlier, we further explored whether we could identify 15 other potential alternate codons that specify the same amino acids in our assays (Figure 4e). A factor is defined to indicate the degree of changes in Cq caused by PNA probe relative to the blank samples, where 31 in the equation corresponded to Cq level of the blank control (Figure 4e). Interestingly, regardless of the introduction of single, two‐adjacent, or several point mutations, all mutant samples obtained large factor values, suggesting that there was no significant impact on amplification with PNA treatment. As for another differential taxa in the heatmap, wild‐type groups depicted factors close to zero since their replications were suppressed to the blank level in the presence of PNA. This result confirmed that PNA‐assisted PCR assays successfully enable identification of SNPs in variants, providing a relative wide range of signal‐to‐noise ratio for accurate identification.

### 2.3 Viral Variants of Concern (VoC) Discrimination via PNA‐Assisted NIR‐Responsive ddPCR

**Figure 5:**
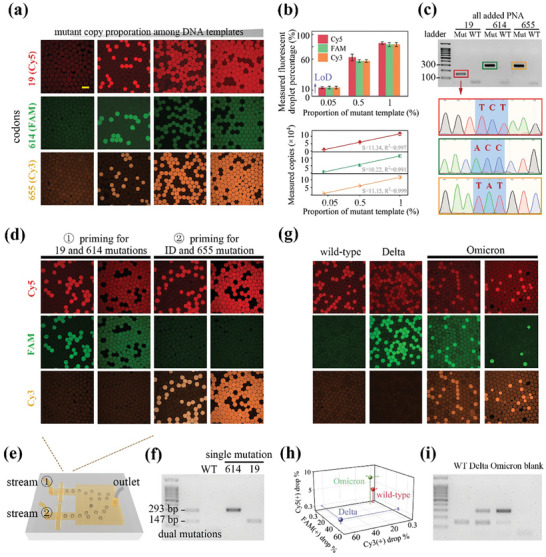
PNA‐assisted photothermal dPCR screening codon 19, 614 and 655 signatures for viral identification and VoC discrimination. a) Confocal fluorescence images showing PNA‐assisted post‐dPCR microcarriers at elevated concentrations of mutant template, using three different probes labeling. b) Observed bright drop percentages and deduced template abundance as functions of the expected amount of mutant‐type in the templates. Error bars, mean ± s.e.m. (*n* = 3). The LoD indicated by the dotted lines represented the mean + two‐fold of the s.e.m. from three negative control tests without DNA. c) Gel electrophoresis and Sanger sequencing analysis of the products derived from the droplets. d) Confocal fluorescence images showing two sets of duplex PNA‐assisted dPCR, detecting two DNA spike concentrations of 0.25 and 2.5 copies per drop, where Cy5, FAM, and Cy3 TaqMan probes were designed to label the presence of mutation at three respective codons. Viral identification was conducted utilizing codon 19′s primers without addition of the PNA. e) The combinational drop‐making chip was employed to pool the emulsions together for irradiation and fluorescence enumeration. f) Gel electrophoresis bands verified the successful detection of existing mutations of interest from the duplex assays. g) Confocal fluorescence images showing color patterns that enabled the differentiation of wild‐type, Omicron, and Delta variants through combinational duplex assays, with Omicron‐type plasmid inputs of 0.8 and 0.3 copies per drop. h) The color coordinates of VoC were divided into different intervals. i) Gel electrophoresis bands from VoC matched their mutation as regulated by PNA‐assisted PCR. Scale bar, 100 µm in (a, d, and g).

Finally, we performed PNA‐assisted NIR‐responsive ddPCR tests for variants discrimination, which converted codons sequence acquisition into triple fluorescent droplet enumerations in dPCR. Detection of dPCR amplification products were facilitated by the specific catalysis preference of TaqMan fluorescent probes, carrying FAM, Cy5, and Cy3 fluorophores for the priming of variations at codons 19, 614, and 655, respectively. As evaluating the capability of photothermal dPCR in detecting SNPs, its specificity and sensitivity at single‐molecule level were simultaneously assessed via a serial dilution experiment (**Figure** **5**a). We spiked mutant DNA into wild‐type DNA in ten‐fold dilution steps, while maintaining total DNA abundance equivalent to 80% of bright droplets before PNA addition. Notably, upon adding PNA, the percentages of post‐dPCR bright droplets reduced to the levels comparable to the abundance of mutant templates. By retrodicting the bright drop counting with Poisson statistics, the estimation of template copies among given number of droplet arrays matched tightly along the serial dilution process (Figure 5b). This result suggests that PNA selectively blocked wild type amplifications, demonstrating consistent effectiveness in the range of 0.05 to 2 average molecules per droplet. Moreover, the limit of detection (LoD) level of 0.3% was defined as the fake bright droplet occurrence in the blank samples, which was consistent with previous reports (Figure 5b; and Table S3, Supporting Information).^[^ ^27^ ^]^ Based on back‐to‐back comparisons, the template amount equivalent to Cq of 22 in qPCR yielded 90% bright droplet results in dPCR. This correspondence was not universal and could be affected by primer efficiency and other factors. As input mutant templates decreased, our droplet‐based digitization method enabled the identification of 8000 copies in the 0.05‐fold diluted sample, representing more than a tenfold improvement compared to qPCR measurement (Figures 4d and 5b; and Table S4, Supporting Information). Successful amplification of mutant templates and blockage of wild‐type templates were further verified by identifying the product bands through agarose gel electrophoresis and Sanger sequencing (Figure 5c; Figure S8, Supporting Information).

In order to simultaneously determine viral presence and discriminate wild‐type, Omicron, and Delta variants, an efficient multiplex assay was exploited to measure SNP signatures in a single experiment with minimum sample consumption. All the primers, PNA, and TaqMan probes (as sequences listed in Table S2, Supporting Information) were divided into 2 duplex panels to address the challenge of screening adjacent codons 614 and 655. In specific, one set of duplex reaction identified codon 19 and 614 mutations, while the other assessed codon 655 and simultaneously performed viral ID using codon 19 assay without a corresponding PNA probe. As proof of concept, the synthetic plasmid carrying mutations of the three codons produced simultaneous red and green fluorescent droplets from the stream 1 reaction. Additionally, this led to a population of droplets emitting red and orange fluorescence from the stream 2 panel (Figure 5d). We carried out this detection with two DNA concentrations, and thus clarified the robust quantitative performance of tracing molecular abundance. In contrast, wild‐type plasmids only generated red fluorescent droplets from the ID reaction and remained negative for the rest of screened regions. Utilizing our designed microfluidic chips (Figure 5e), the two reaction streams were separately emulsified and collected together for analysis. Later, the gel bands of 147 and 293 bp from electrophoresis demonstrated multiple mutant DNA target products were generated as expected, along with fluorescence signals in droplets (Figure 5f).

Three types of plasmids containing the spike protein gene with particular mutations were constructed to simulate the virus and were subjected to our detection assay (Figure 5g; and Table S5, Supporting Information). As expected, the results obtained from wild‐type and Delta‐type plasmids yielded single red and dual red‐green colors, respectively. Conversely, Omicron‐type plasmids featured three color fluorescent drops, with green and orange positive signals deriving from the same portion of droplets. The detection results of gradient template concentrations (5100 and 23600 copies) confirmed that this droplet‐based digitization method can accurately and quantitatively reflect the abundance with a 5‐fold difference in resolution. This was achieved without being affected by the primer sets efficiency or the need for standard curves in the qPCR method. As a general characterization of all assays, the measured concentration of DNA matched the anticipated concentration within the range of 5% to 83% of bright drop percentages. Additionally, the coordinates of color code were identifiable and consistent with the mutation conditions (Figure 5h–i). In short, these results demonstrated the feasibility and specificity of the PNA‐assistant photothermal dPCR strategy for rapid and quantitative multisite SNPs identification.

## 3 Conclusion

In summary, we have introduced a novel technique combining multiplexed NIR photothermal dPCR and PNA clamp‐based biochemical characterization to profile triple SNPs for the diagnosis of SARS‐CoV‐2 infection and identification of viral variants of concern. By optimizing shell thickness and other parameters, we have successfully developed highly responsive homogeneous microcarriers. Compared to existing droplet techniques, our intelligent‐responsive microcarriers alleviate evaporation issues, exhibit enhanced thermal stability, and provide accurate NIR‐induced temperature cycle customization capability. The integration of sequence‐recognized bioreactions has further advanced the capability of our technique for complex point‐of‐care testing.

We have also developed a vacuum suction‐based droplet generation device for microfluidic chips and a programmable NIR control module to automatically complete the entire process. Microfluidic microcarriers are particular suitable for multiplex assays, as each droplet contains multiplexed reagents while primarily confining one single DNA at most. In this way, amplification competition and interference between DNA templates are greatly avoided, enabling signal resolution at the single‐molecule level.

Our experiments have validated the effectiveness of PNA clamps in selectively amplifying mutant copies and achieving single‐base recognition resolution. Due to the quantitative and multiplexed nature of dPCR, we have successfully demonstrated the simultaneous PNAs‐clamp based detection of multiple targets, including adjacent mutation sites, by categorizing them into parallel panels. The results of the triple‐color fluorogenic labeling are in complete agreement with genotypes, and the detection sensitivity has been improved by more than 10‐fold.

Taken together, these features paint a promising outlook that PNA‐assisted photothermal dPCR technologies, powered by intelligent and biochemically compatible microcarriers, could become valuable diagnostic tools for diseases influenced by SNPs.

## 4 Experimental Section

### Materials

Tris buffer, dopamine hydrochloride, Tween 20, and Gelatin (G7041) were obtained from SIGMA. Pyridine, tetraethoxysilane, hydrochloric acid, and pure anhydrous ethanol were purchased from Xilong Scientific and Sinopharm Chemical. PCR reaction kit, primers, PNA, TaqMan probes, and GO dispersion solution were supplied from ABclonal (RK20602), Shanghai Shenggong, Tanzhen Bio, Shanghai Generay, and XFNANO Materials Tech., respectively. Polydimethylsiloxane, fluoroSurfactant, and HFE 7500 inert oil were purchased from SYLGARD, RAN Biotech., and 3 m Novec respectively. UNO Ardunio chip, 0–10 V voltage amplifier, power supply, and fan sets were purchased from Seeedstudio, SLQXF, Wintech, and Studing, respectively. Deionized water used in this study was obtained from the Millipore Milli‐Q Plus 185 system.

### Morphology Characterization

The morphology characterization, elemental mapping, and size distribution of nanomaterials were characterized by SEM (SU8010), TEM (FEI Tecnai G2 F20), and Nanophox Zetasizer (ZEN3600). Prior to imaging, sample surfaces were sprayed with platinum to improve electroconductibility. Acceleration voltage and magnification of SEM measurement were selected ranging at 5–10 kV and 10–200 k folds, respectively. These parameters of TEM were set ≈100–200 kV and 10–100 k folds, respectively.

### Properties Characterization

Infrared spectra were measured by FTIR Spectrometer (Tensor II). Moduli of gel cubes were characterized as a function of rising temperature by DHR‐2 Rheometer, using strain and frequency settings of 0.1% and 1 Hz, respectively. Strain properties of gel cubes were measured by Instron 5944 mechanical testing system, using pulling speed of 0.1 mm s^−1^.

### Microfluidic Operation

Droplet generation process was conducted using self‐assembled vacuum‐based setup. PCR reaction and HFE 7500 oil were loaded in pipette tips and introduced to the co‐flow junctions to produce microcarriers. Fluorescent imaging of post‐PCR microcarriers was performed by Nikon A1laser scanning confocal microscope with acquisition in FAM, Cy5, Cy3 channels, and bright field. A diode 808 nm NIR laser source, UNO Arduino circuit, power supply, linear amplifier, and fan set were self‐assembled together for automated irradiation. An infrared thermal imaging camera was used to monitor the real‐time temperature progression of the sample.

### Synthesis of Fe_3_O_4_@GO@SiO_2_

The aforementioned Fe_3_O_4_ NPs were first encapsulated with GO nanosheet through the conglutination of polydopamine. According to the previous work, 10 mL of reaction sample contained 2 mL of Fe_3_O_4_ NPs dispersion (≈0.5 g mL^−1^), 1.5 mL of dopamine hydrochloride (13.3 mg mL^−1^), 6.5 mL of Tris·HCl buffer (pH 8.5) was shaken for 6 h in an open glass vessel. At that point, the polydopamine coated on the NPs and turned the solution color to black. After centrifuging and discarding the supernatant, the NPs were ultrasonically dispersed in 10 mL of GO dispersions (0.8 mg mL^−1^) and constantly stirred for 6 h to obtain the Fe_3_O_4_@GO composite. After washing thrice with water, the composite was deposited with a silica shell by the modified Stöber method to obtain the desired Fe_3_O_4_@GO@SiO_2_.

### FITC Labeling of the Nanocomposite

FITC dye was bonded to the silica surface via amine groups. FITC (10 mg) was first reacted with 100 µL of aminopropyltriethoxysilane (APTES) in 5 mL of ethanol for one day. Then the nanocomposite was added and stirred for another 24 h in the dark. Finally, the FITC‐grafted nanocomposite was washed several times to remove unattached FITC molecules.

### Photothermal Digital PCR

DNA plasmids containing spike protein gene were synthesized and transformed into engineering *Escherichia coli* by Shanghai Shenggong Company. The plasmids DNA were duplicated by culturing bacteria in LB media and then were extracted and eluted into 60 µL water by Plasmids DNA cleanup kit (Qiagen). Then 1–10 µL cDNA was introduced into a 50 µL PCR reaction solution containing 5% of gelation, 0.3 µm of primers and TaqMan probe, 0.5 µL of BSA and Tween 20, and the nanocomposite. A PDMS‐based flow‐focusing microfluidic device with different dimension was fabricated to split the 50 µL solution into gelatin microcarriers in HFE‐7500 oil. Driven by the Arduino program, typical PCR thermal cycles were performed as 95 °C for 3 min, 33 cycles of 95 °C for 30 s, 75 °C for 60 s, 57 °C for 60 s, 72 °C for 60 s. Real‐time PCR, PCR, and gel electrophoresis were conducted by Roche (LightCycler 96) and BioRad instruments. Finally, the proportion of fluorescent microcarriers was enumerated under laser confocal scanning microscope. All samples were tested in triplicate.

### Statistical Analysis

All quantitative characterizations were presented as means with standard deviations (*n* = 3). p‐values were obtained using one‐way ANOVA with Tukey's multiple comparisons test. Statistical significance was defined as p ≤ 0.05, and the values were labeled with ** for p ≤ 0.01 and *** for p ≤ 0.001.

## Conflict of Interest

The authors declare no conflict of interest.

## Author Contributions

L.X.Z., P.R., S.Y.L., M.S.C., and R.X.Z. conducted experiments and data analysis; L.X.Z. wrote the manuscript with the help from S.Y.L., Q.H.F., and F.F.Y.

## Supporting information

Supporting Information

## Data Availability

The data that support the findings of this study are available from the corresponding author upon reasonable request.
