# Supplementary material for: Peptide Nucleic Acid Clamp‐Assisted Photothermal Multiplexed Digital PCR for Identifying SARS‐CoV‐2 Variants of Concern
Source: Adv Sci (Weinh). 2024 Jan 19;11(13):2306088. doi: 10.1002/advs.202306088 (PMC10987151; doi:10.1002/advs.202306088)

ADVANCED SCIENCE
Open Access

Supporting Information

for *Adv. Sci.*, DOI 10.1002/advs.202306088

Peptide Nucleic Acid Clamp-Assisted Photothermal Multiplexed Digital PCR for Identifying SARS-CoV-2 Variants of Concern

Lexiang Zhang, Rokshana Parvin, Siyue Lin, Mingshuo Chen, Ruixuan Zheng, Qihui Fan and Fangfu Ye**

Supplementary Information for

Peptide Nucleic Acid Clamp-assisted Photothermal Multiplexed Digital PCR for Identifying SARS-CoV-2 Variants of Concern

Lexiang Zhang, Rokshana Parvin, Siyue Lin, Mingshuo Chen, Ruixuan Zheng, Qihui Fan*, Fangfu Ye*

Corresponding author: Qihui Fan, Fangfu Ye

Email: fanqh@iphy.ac.cn; fye@iphy.ac.cn

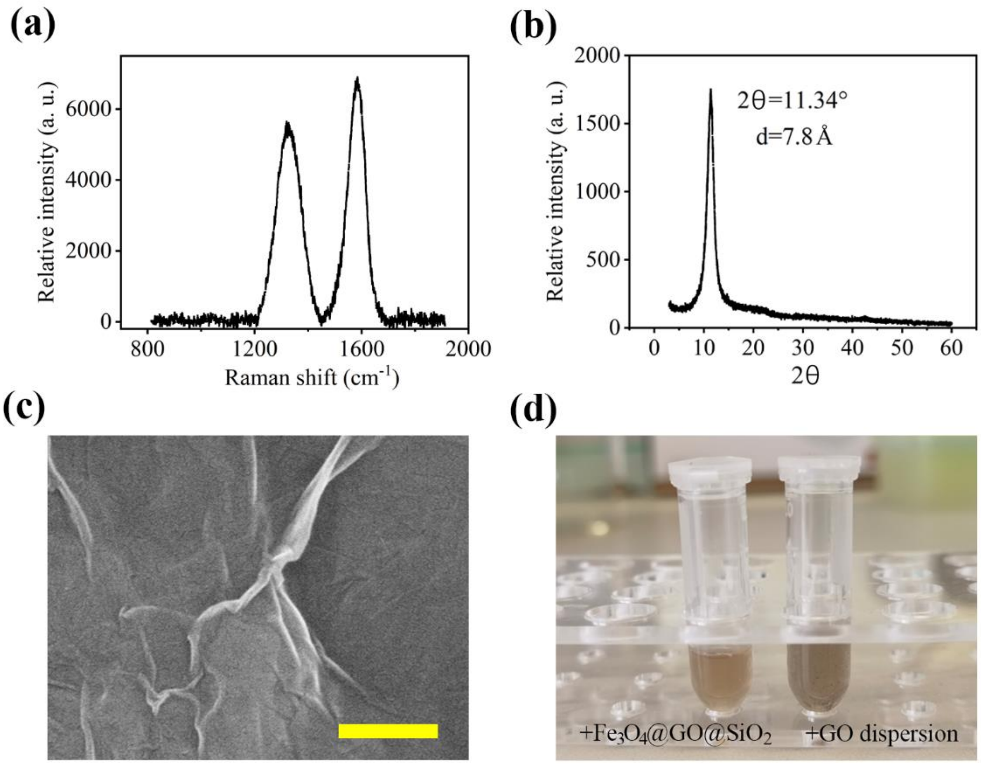

Figure S1. Morphology and physicochemical characteristic of GO nanosheets. (a) Raman spectra of graphene oxides showing characteristic peaks centered at ~1350 and 1585 cm^{-1} respectively. (b) XRD analysis. (c) SEM image. (d) Photograph of the dispersion of GO nanosheet and Fe_3O_4@GO@SiO_2 in 10 % gelatin samples, respectively, after a few rounds of heating to 95 ℃. Scale bar, 250nm in (c).

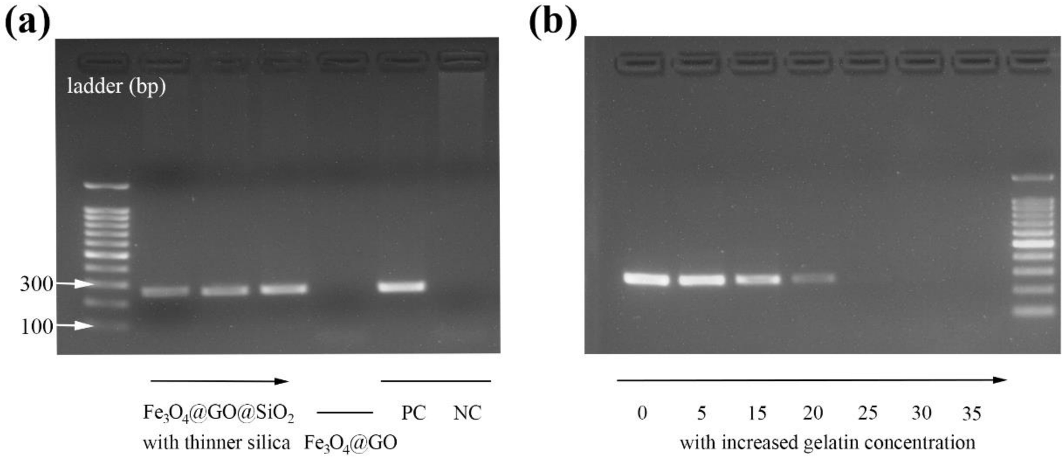

Figure S2. Electrophoresis gel images showing biocompatibility of nanocomposite-doped gelatin substrate for PCR. PC and NC columns represent positive and negative controls without nanomaterial addition. PCR efficiency as functions of (a) silica shell thickness and (b) gelatin concentration.

Table S1. Size distributions with the polydispersity indexes and standard deviations

	**diameter**	**polydispersity index**	**standard deviation**
Fe_3O_4@GO	335	0.213	146.6
Fe_3O_4@GO@SiO_2 (with four different silica thickness)	1313	0.335	365.3
	1235	0.542	236.5
	940	0.322	312.7
	742	0.314	246.9

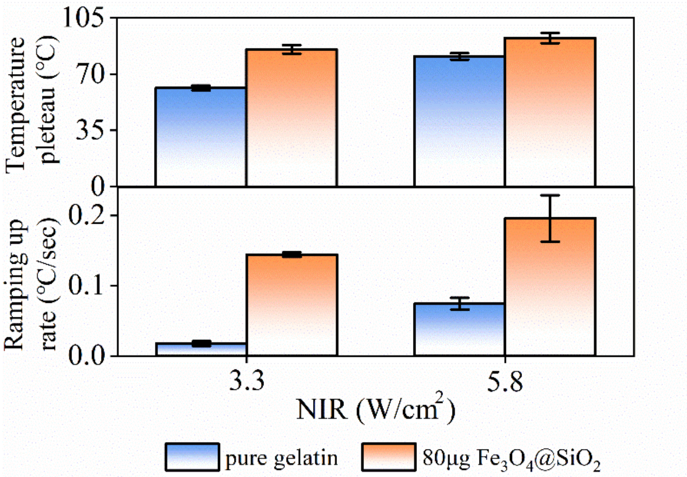

Figure S3. Temperature climbing evaluation of 50 μL gelatin samples with and without $Fe_3O_4@SiO_2$ NPs addition. Error bar, mean ± s.e.m. (n= 3).

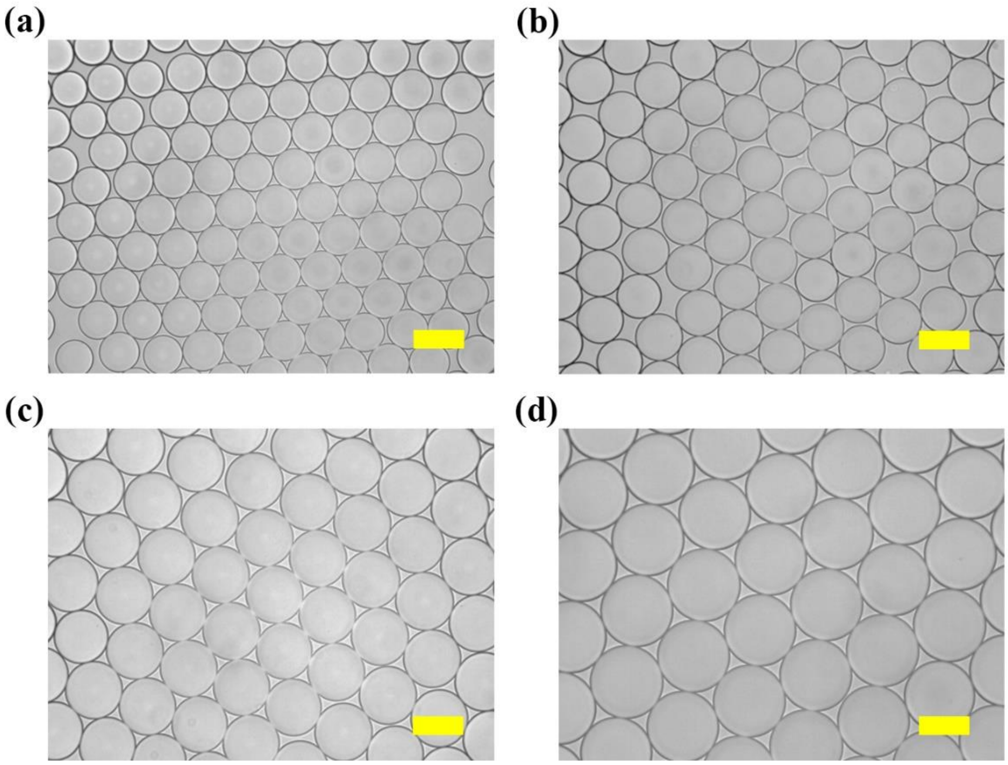

Figure S4. Uniformity of produced microcarriers with average diameter of 83, 95, 116, and 126 μm, respectively. Scale bar, 100 μm.

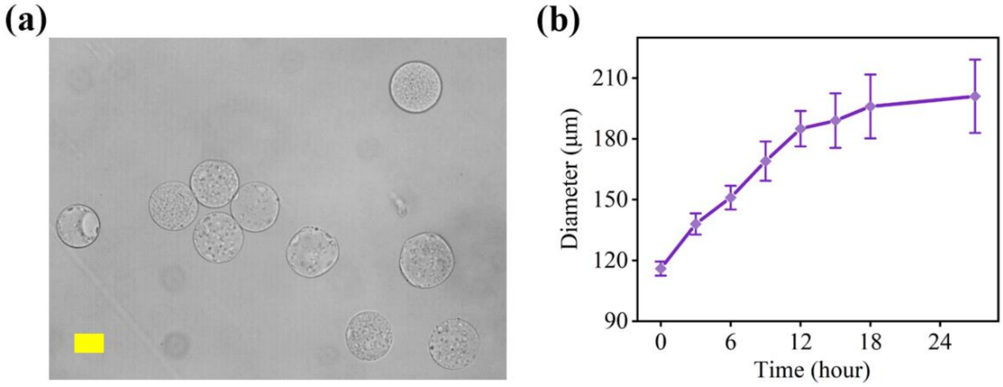

Figure S5. Characterization of microcarriers' swelling behavior. (a) Microcarriers with a diameter of 116 μm exhibited a homogeneous size and swelled by 72% over a period of 27 hours. (b) Swelling curves in aqueous solution plotted as a function of time. Scale bar, 100 μm. Error bars, mean ± s.e.m. (n= 3).

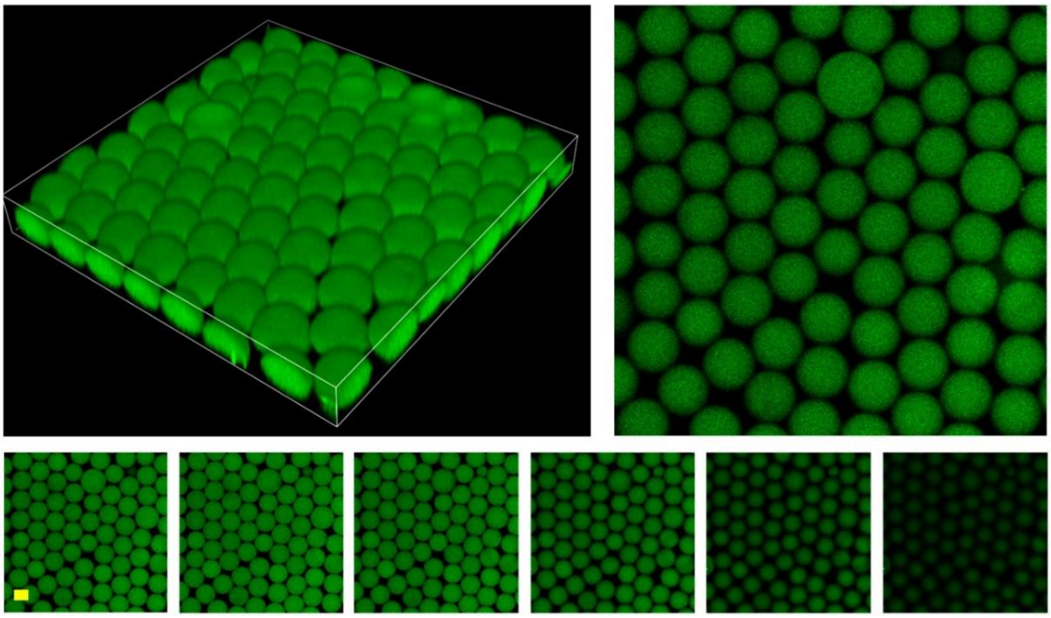

Figure S6. A broad field of fluorescence confocal view including a few dozens of microcarriers containing FITC-labelled nanocomposite and a series of slices acquired using the same z-scanning step. Scale bar, 100 μm.

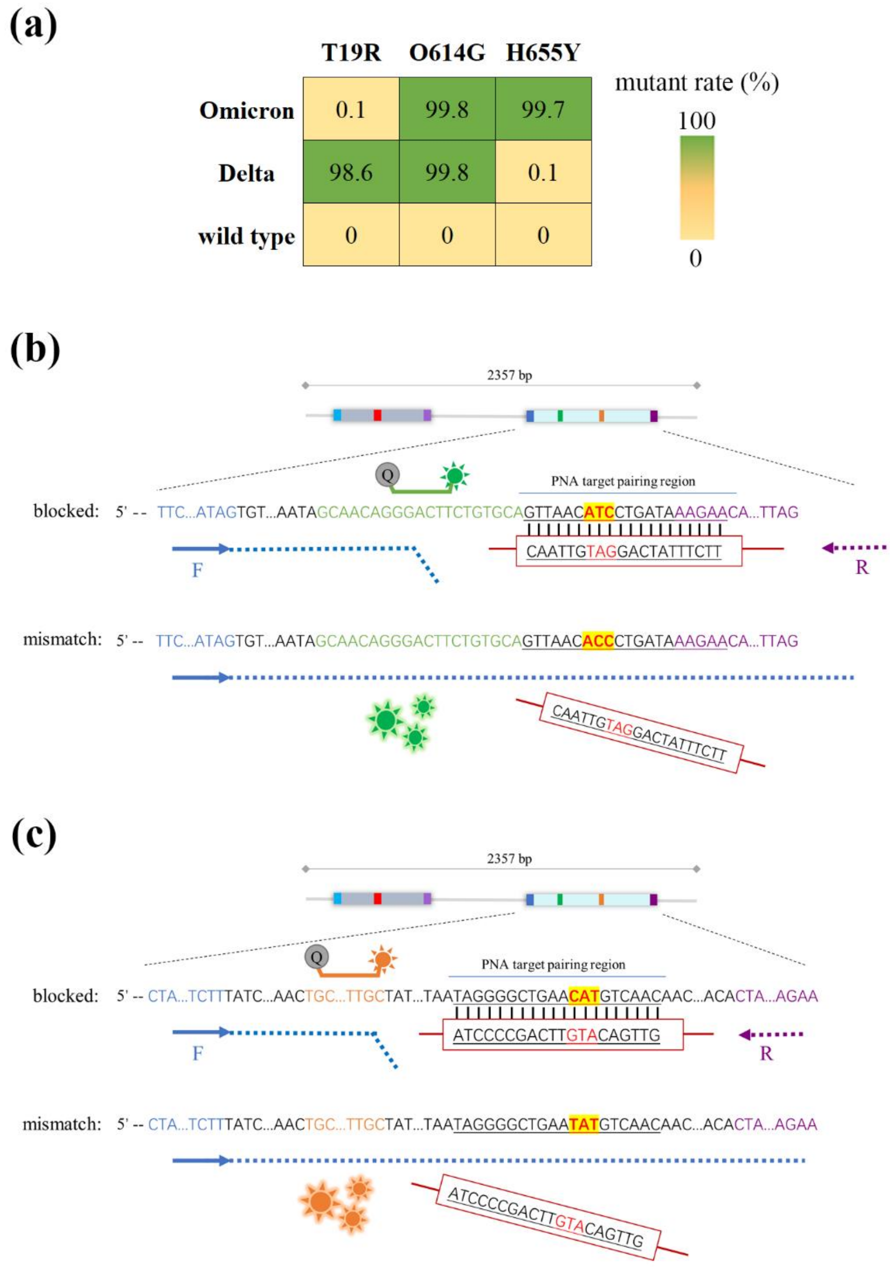

Figure S7. (a) Highly conserved SNP signatures used for distinguishing variants of interest based on official reported lineage comparisons. (https://outbreak.info/) Schematic of detecting (b) O614G and (c) H655Y mutations within SARS-CoV-2 spike protein gene. Strategy of designing PNA to selective block wild-type sequence and allow fluorescence signal only derive from targeted mutations. The sequences and positions of the targeted codons, PNA probes, primers, and TaqMan probes are indicated.

Table S2. Sequencing of designed PNA clamps, primers and TaqMan probes. 19-2 stands for the second design version targeting at codon 19 of the spike protein gene.

Name	Sequences (starting from 5')
PNA-614	TTCTTTATCAGGATGTTAAC
PNA-19	TTAATCTTACAACCAGAACT
PNA-655	AGTTGTTGACATGTTCAGCC
19-forward primer	GCCACTAGTCTCTAGTCAG
19-reverse primer	GGTAAGAACAAGTCCTGAG
19-TaqMan (Cy5)	CACACGTGGTGTTTATTACCCTGAC
614-forward primer	CTAACCAGGTTGCTGTTCTT
614-reverse primer	TTCTGCACCAAGTGACATAG
614-TaqMan (FAM)	TGCACAGAAGTCCCTGTTGC
655-forward primer	CTAACCAGGTTGCTGTTCTT
655-reverse primer	TTCTGCACCAAGTGACATAG
655-TaqMan (Cy3)	TGCACAGAAGTCCCTGTTGC
PNA-19-v2	TGTGTTAATCTTACAACCAG
PNA-19-v3	GTTCTGGTTGTAAGATTAAC
PNA-19-v4	TGAGTTCTGGTTGTAAGATT
PNA-655-v2	GTTGACATGTTCAGCCCCTA
PNA-655-v3	CTGAACATGTCAACAACTCA
PNA-19-fv	TTCTGGTTGTAAGATTAA
PNA-655-fv	TGTTGACATGTTCAGCC

* v2 and fv represent version 2 of the other available designs and failed version, respectively.

Table S3. Significance analysis of different groups in Figures 5b, where "ns" represents not significant.

fluorescent channel	mutant template %	Cy5			FAM			Cy3		
		0.05	0.5	1	0.05	0.5	1	0.05	0.5	1
Cy5	0.05		***	***	ns	***	***	ns	***	***
	0.5			***	***	ns	***	***	ns	***
	1				***	***	ns	***	***	ns
FAM	0.05					***	***	ns	***	***
	0.5						***	***	ns	***
	1							***	***	ns
Cy3	0.05								***	***
	0.5									***
	1									

Table S4. Back-to-back comparison with the bulk qPCR approach based on the assay priming codon 19.

DNA template input (×10^4 copy)	Bulk qPCR (Ct)		dPCR (bright drop %)	
	average	SD.	average	SD.
19.4	23.8	1.7	93.0	1.7
11.6	25.5	1.6	81.7	1.2
5.5	26.8	0.8	58.7	3.1
0.6	30.8	1.2	12.7	1.5
0 (blank)	32.7	1.0	0.2	0.1

* DNA template inputs were deduced from bright drop percentage in dPCR assay.

* The LoD of bulk qPCR was around 5×10^4 copy, beyond which the Cq was indistinguishable from the blank control.

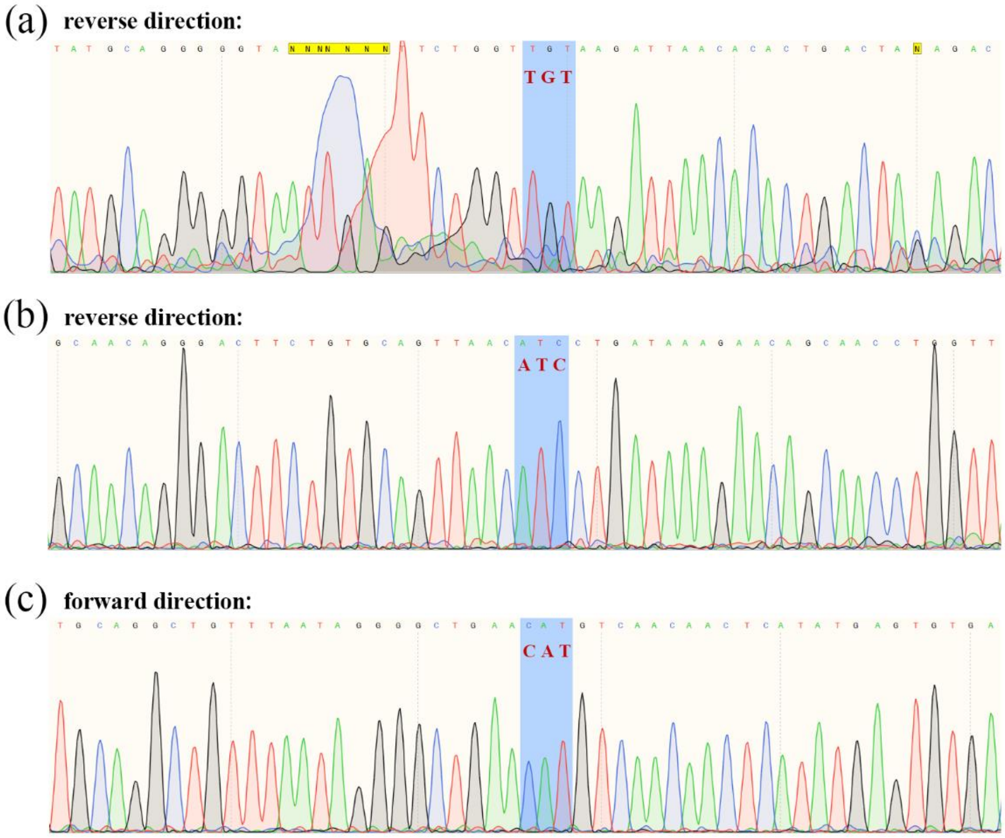

Figure S8. Sanger sequencing analysis of the NIR-responsive dPCR products harboring the wild type reads at codon (a) 19, (b) 614 and (c) 655.

Table S5. Synthetic plasmid containing the sequence of spike protein gene.

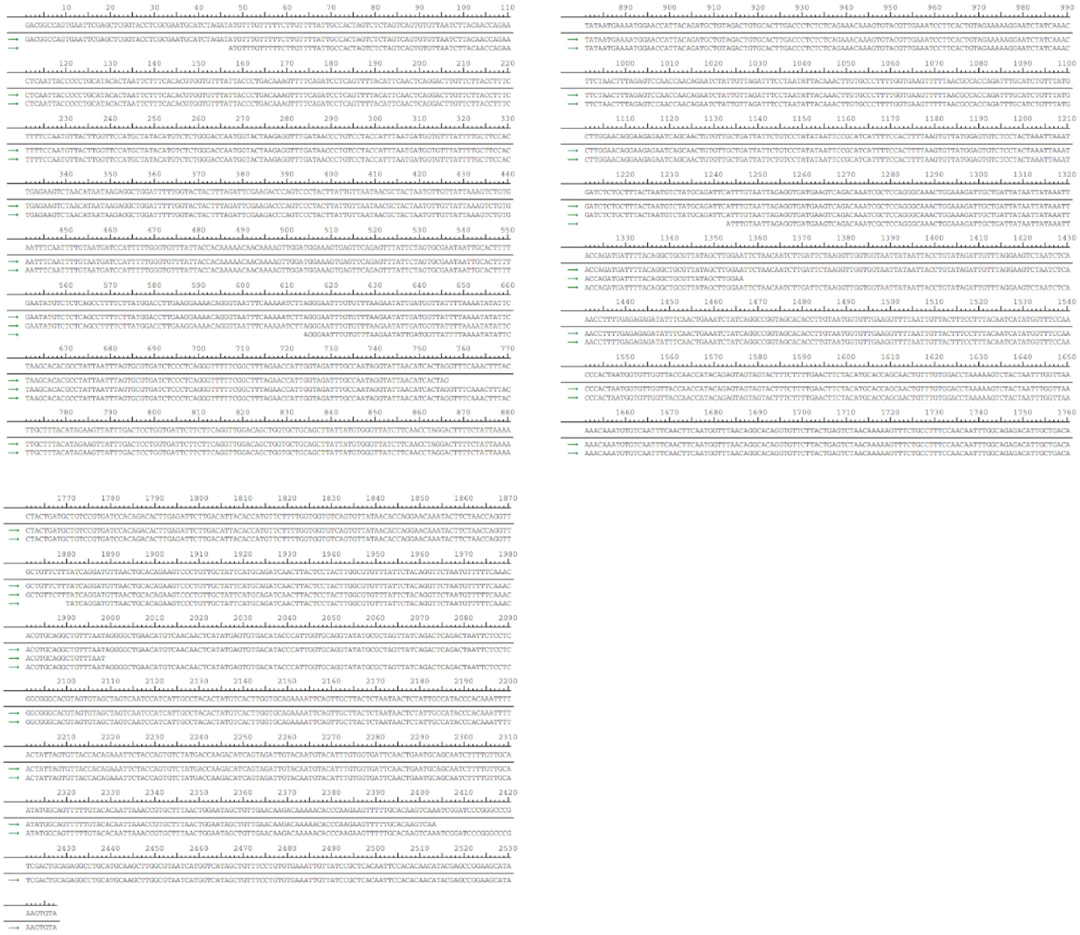

Supplement: Supplementary file 1 — Supporting Information [file ADVS-11-2306088-s001.pdf]
